# Genomic analysis of a spontaneous unifoliate mutant reveals gene candidates associated with compound leaf development in *Vigna unguiculata* [L] Walp

**DOI:** 10.1038/s41598-024-61062-x

**Published:** 2024-05-09

**Authors:** Offiong Ukpong Edet, Benjamin Ewa Ubi, Takayoshi Ishii

**Affiliations:** 1 Arid Land Research Center, Tottori University, Tottori, 680-0001 Japan; 2 Department of Crop Science, University of Calabar, PMB 1115, Calabar, Cross River State Nigeria; 3 Department of Biotechnology, Ebonyi State University, Abakaliki, Ebonyi State Nigeria

**Keywords:** Computational biology and bioinformatics, Genetics, Molecular biology, Plant sciences

## Abstract

Molecular mechanisms which underpin compound leaf development in some legumes have been reported, but there is no previous study on the molecular genetic control of compound leaf formation in *Vigna unguiculata* (cowpea), an important dryland legume of African origin. In most studied species with compound leaves, class 1 *KNOTTED-LIKE HOMEOBOX* genes expressed in developing leaf primordia sustain morphogenetic activity, allowing leaf dissection and the development of leaflets. Other genes, such as, *SINGLE LEAFLET1* in *Medicago truncatula* and *Trifoliate* in *Solanum lycopersicum,* are also implicated in regulating compound leaf patterning. To set the pace for an in-depth understanding of the genetics of compound leaf development in cowpea, we applied RNA-seq and whole genome shotgun sequence datasets of a spontaneous cowpea unifoliate mutant and its trifoliate wild-type cultivar to conduct comparative reference-based gene expression, de novo genome-wide isoform switch, and genome variant analyses between the two genotypes. Our results suggest that genomic variants upstream of *LATE ELONGATED HYPOCOTYL* and down-stream of *REVEILLE4*, *BRASSINOSTERIOD INSENSITIVE1* and *LATERAL ORGAN BOUNDARIES* result in down-regulation of key components of cowpea circadian rhythm central oscillator and brassinosteroid signaling, resulting in unifoliate leaves and brassinosteroid-deficient-like phenotypes. We have stated hypotheses that will guide follow-up studies expected to provide more insights.

## Introduction

Morphogenetic variation between simple and compound leaf forms begin with the developing leaf primordia on the peripheral zone of shoot apical meristem (SAM), and the expression of class 1 *KNOTTED-LIKE HOMEOBOX* (*KNOXI*) genes in developing leaf primordia is thought to play a defining role in this variation^1–4^. Unraveling the molecular mechanisms controlling the differences in simple and compound leaf morphogenesis has a long history and has remained relevant, following the flexibility in the molecular events that result in diverse leaf patterns^5–9^. The phases of leaf ontogenesis—initiation, primary morphogenesis, and secondary morphogenesis—are similar in simple and compound leaves^2,10–12^. However, the formation of separated leaflet primordia in species with compound leaves, based on which leaflet number is determined, distinguishes compound and simple leaf primary morphogenesis^3^. The distinguishing pattern of expression of the *KNOXI* genes in tomato, in comparison with *KNOXI* expressions in species with simple leaves, established the molecular basis for differentiating the development of simple and compound leaves^9^. Efforts to investigate the mechanisms of compound leaf development in legumes have implicated other genes in the regulation of compound leaf patterning. While *KNOX1* genes are absent in the developing leaf primordia of *Medicago truncatula* and *Pisum sativum*, legumes of the inverted repeat-lacking clade (IRLC), legume-specific *FLORICAULA/LEAFY* orthologs, *SINGLE LEAFLET1* and *UNIFOLIATA*, respectively, regulate compound leaf development in the two legume species^13,14^. Conversely, *SHOOT MERISTEMLESS*-like *KNOX* genes are associated with compound leaf development in *Vigna radiata*^15^, suggesting that absence of *KNOXI* genes expression in leaf primordia of some legumes species may be restricted to legumes in the IRLC^15–18^. In soybean, the legume-specific transcription factor, *E1*, earlier reported to regulate flowering and maturation^19–21^, is also associated with leaf development^22^; however, there is no evidence which associates *E1* with compound leaf formation in soybean.

Cowpea (*Vigna unguiculata*; 2n = 2x = 22) is an economically important legume crop^23,24^, which produces two opposite simple leaves as the first pair of leaves, and subsequently bears alternate trifoliate compound leaves. Genetic inheritance of unifoliate mutant loci in a natural unifoliate mutant^25^ and unifoliate mutant segregants in F_2_ and advanced generations of crosses involving wild-type trifoliate cowpea cultivars^26^ have been studied, but the underlying molecular mechanisms of cowpea leaf development has not been previously reported. Here, using shotgun sequences and RNA-seq datasets of a spontaneous unifoliate cowpea mutant and its wild-type variety, we combined reference-based differential gene expression, de novo genome-wide isoform switch (IS), and genome variant analyses to identify phenotype-causing candidate genes and significantly enriched or depleted Gene Ontology (GO) terms associated with the mutant. Our study suggests that nucleotide variations upstream of *LATE ELONGATED HYPOCOTYL* (*LHY*) and down-stream of *REVEILLE4* (*RVE4*), *BRASSINOSTEROID INSENSITIVE 1* (*BRI1*) and *LATERAL ORGAN BOUNDARIES* (*LOB*) repress the components of cowpea circadian rhythm central oscillator and brassinosteroid (BR) signaling, resulting in the development of unifoliate leaves and phenotypes associated with BR deficiency^27,28^. This serves as a foundation for further research towards elucidating the genetic control of compound leaf patterning in cowpea.

## Results

### Genetic characterization of a novel natural dominant mutant in cowpea

**Figure 1:**
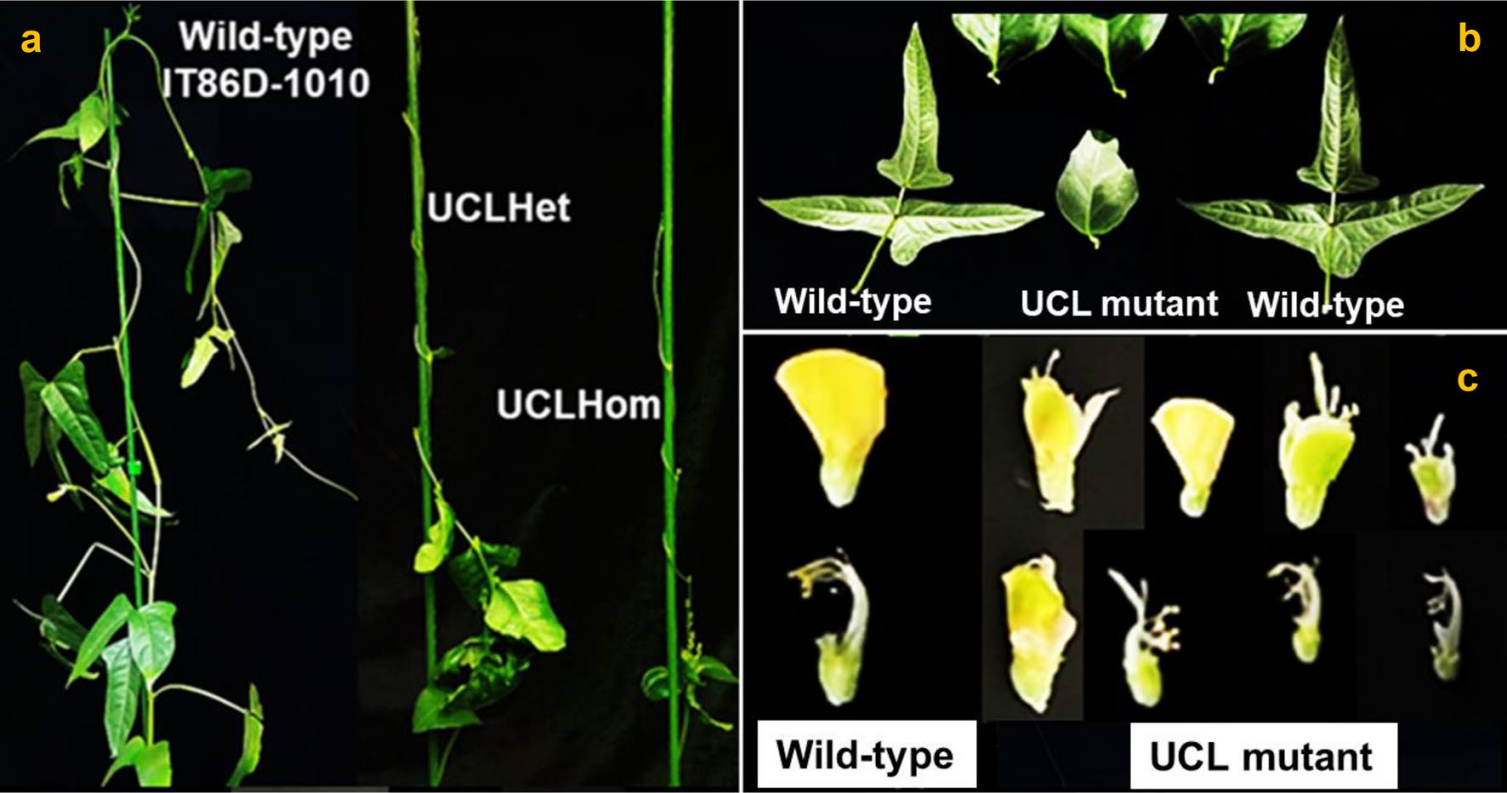
Morphological variation between cowpea wild-type (IT86D-1010) and UCL mutant. (**a)** Wild-type, UCLHet and UCLHom plants; **(b)** Wild-type and UCL mutant leaves; **c.** Wild-type and UCL mutant flowers; UCL, unifoliate curled leaf, UCLHet, UCL heterozygous mutant; UCLHom, UCL homozygous mutant.

We identified a spontaneous mutant bearing subsessile dark green unifoliate curled leaves (UCL) with elongated petiole-like pulvini (Fig. 1a, b) in the 2021 cowpea experimental field of Arid Land Research Center (ALRC), Tottori University, Japan. The mutant arose as a spontaneous segregant from the self-pollinated progeny of a cowpea wild-type variety, IT86D-1010 (Fig. 1a). Compared with the wild-type variety, the mutant plants (heterozygotes and homozygotes) are smaller, bear more structurally deformed than normal flowers (Fig. 1a,c), and yield significantly (p < 0.01) less shoot biomass and pods (Table 1). Production of abnormal flowers by cowpea unifoliate mutants is not unusual^25,26^, but the underlying molecular mechanism is still unclear. The low pod yield is largely caused by the obvious reduced self-pollination resulting from the structural deformity of the flowers, especially the protrusion and physical separation of stamens from the pistil of some flowers (Fig. 1c). Our observation of six self-pollinated generations of the mutant (heterozygote, homozygote and revertant) revealed that the mutant phenotype consistently expresses in both heterozygotes and homozygotes, with segregation in the progenies of heterozygotes conforming to the expected Mendelian 1:2:1 ratio for complete dominance; no segregation was observed in the progenies of homozygous mutant and revertant plants (Table 2). Also, reciprocal crosses between the mutant plants and three wild-type cowpea varieties (Sasaque, IT97K-499–35 and IT86D-1010) confirmed that the mutant locus is dominant. Crosses involving homozygous mutant plants (UCLHom) and the wild-type cowpea varieties produced only heterozygous mutant plants (UCLHet), while the progenies of crosses between UCLHet plants and the wild-type cowpea varieties segregated in approximately 1:1 ratio, UCLHet:Revertant (Table S1).

**Table 1:** Agro-morphological variations between IT86D-1010 and UCL mutant (Mean ± SEM).

Genotype	Leaf type	Total shoot biomass (g)	Days to 50% flowering)	Pod length (cm)	No. of pods/plant	No. of seeds/pod	100 seed weight (g)
IT86D-1010	trifoliate	109.3 ± 0.99^a^	40.2 ± 0.31^a^	15.6 ± 0.28^a^	7.0 ± 0.15^a^	10.8 ± 0.28^a^	15.4 ± 0.04^a^
UCLHet	unifoliate	37.2 ± 0.45^b^	39.8 ± 0.40^a^	8.3 ± 0.70^b^	4.0 ± 0.35^b^	5.5 ± 0.55^b^	15.4 ± 0.02^a^
UCLHom	unifoliate	25.6 ± 0.42^c^	40.0 ± 0.26^a^	7.8 ± 0.59^b^	2.0 ± 0.16^c^	5.2 ± 0.55^b^	15.4 ± 0.05^a^

UCL, unifoliate curled leaf; Het, heterozygote; Hom, homozygote; SEM, standard error of the mean. Means with the same letter in the same column are not significantly different (p > 0.05). Means with different letters in the same column are significantly different (p < 0.01).

**Table 2:** UCL locus segregation in six self-pollinated generations.

Generation	Progeny size (heterozygote)	UCLHet			Generation	Progeny size (homozygote)	UCLHom	Revertant
		Homozygote	Heterozygote	Revertant				
F_1_	24	5	13	6	F_1_	50	50	50
F_2_	200	54	98	48	F_2_	50	50	50
F_3_	200	43	104	53	F_3_	50	50	50
F_4_	200	51	99	50	F_4_	50	50	50
F_5_	200	46	103	51	F_5_	50	50	50
F_6_	200	52	97	49	F_6_	50	50	50

UCL, unifoliate curled leaf; Hom, homozygote; Het, heterozygote. From F_2_, the same number of randomly sampled seeds from each genotype were sown and observed as progeny families, and the segregants from all the progeny families/genotype were summed up to determine mutant locus segregation. In all the generations, there was no segregation in the progenies of UCL homozygous and revertant plants. F_1_ here refers to the first self-pollinated generation after identification of the mutant in the field; self-pollinated progenies of UCLHom and revertant were observed from F_2_, after they were identified in F_1_.

### Sequencing quality and reads mapping metrics

We conducted two categories of RNA-seq-based differential gene expression analyses between UCLHom and IT86D-1010: reference-based differential gene expression analysis based on normalized gene counts, and genome-wide differential isoform usage (DIU) analysis, using de novo assembled transcripts. As the mutant allele is dominant and the phenotypic difference between UCLHet and UCLHom is obvious (Fig. 1a), UCLHom plants used for preparation of RNA-seq libraries were selected from confirmed non-segregating self-reproducing progeny of UCLHom (Table 2). Strand-specific paired-end poly(A)-selected RNA-seq libraries of two bulked biological replicates, composed of 20 plants each of the purified RNA samples of UCLHom and IT86D-1010 were prepared using TruSeq Stranded mRNA Library Prep Kit (Illumina). The libraries were sequenced with DNBSEQ-G400 (MGISEQ-2000RS) short reads sequencer (Data S1 – S8). Initial read quality assessment was done with *FastQC* (https://www.bioinformatics.babraham.ac.uk/projects/fastqc/), after which the sequences were processed by *fastp*^29^. Both *FastQC* and *fastp* results indicated sequencing adequacy (See Figure S1a and S1b; Table S2). The *fastp* results showed 10.7 to 13 Gb/library, 109 – 133 M reads/library, and average read length of 100 b (See Table S2). *RNA STAR* mapper^30^ was applied to align the ***fastp***-filtered sequences to *Vunguiculata_540_v1.0* reference genome, with *Vunguiculata_540_v1.2* reference annotation as the gene model (https://data.jgi.doe.gov/refine-download/phytozome?genome_id=540). A multi-sample 2-pass mapping was conducted to enable accurate discovery and quantification of splice junctions^31^. Post-alignment RNA-seq-specific quality metrics aggregated with ***MultiQC***^32^ confirmed the suitability of the datasets for the intended downstream applications. Importantly, in all the samples, over 80% of the reads were uniquely mapped, and more than 80% of the mapped reads mapped on CDS exons. Gene coverage plot showed no bias, and junction saturation plots showed stability of known splice junctions, indicating saturated sequencing depth (See Figure S2)^33^.

We used UCLHom, UCLHet and wild-type revertant F_2_ segregants of IT97K-499–35 × UCLHom hybrid to map candidate mutations in the UCL mutant genome. The genome sequences of the UCL mutant bulk (bulked DNA samples of 10 plants each of UCLHet and UCLHom) and wild-type revertant (bulked DNA samples of 20 revertant plants) generated by DNBSEQ-G400 short reads sequencer (Data S9 – S12) were analyzed by ***FastQC***. The results, which showed phred scores of more than 30 (See Figure S1c and S1d), indicated sequencing adequacy. However, we still trimmed the sequences with ***fastp*** before applying ***Bowtie2***^34^ to align the short reads to *Vunguiculata_540_v1.0* reference genome. The ***fastp***-filtering results showed an average read length of 150 b in the two libraries, about 295.2 M sequence reads and 44 Gb in the revertant bulk library, and 365.8 M reads and 54.7 Gb in the UCL mutant bulk library (See Table S2).

### Reference-based differential gene expression and functionally enriched or depleted GO terms in the UCL mutant genome

**Figure 2:**
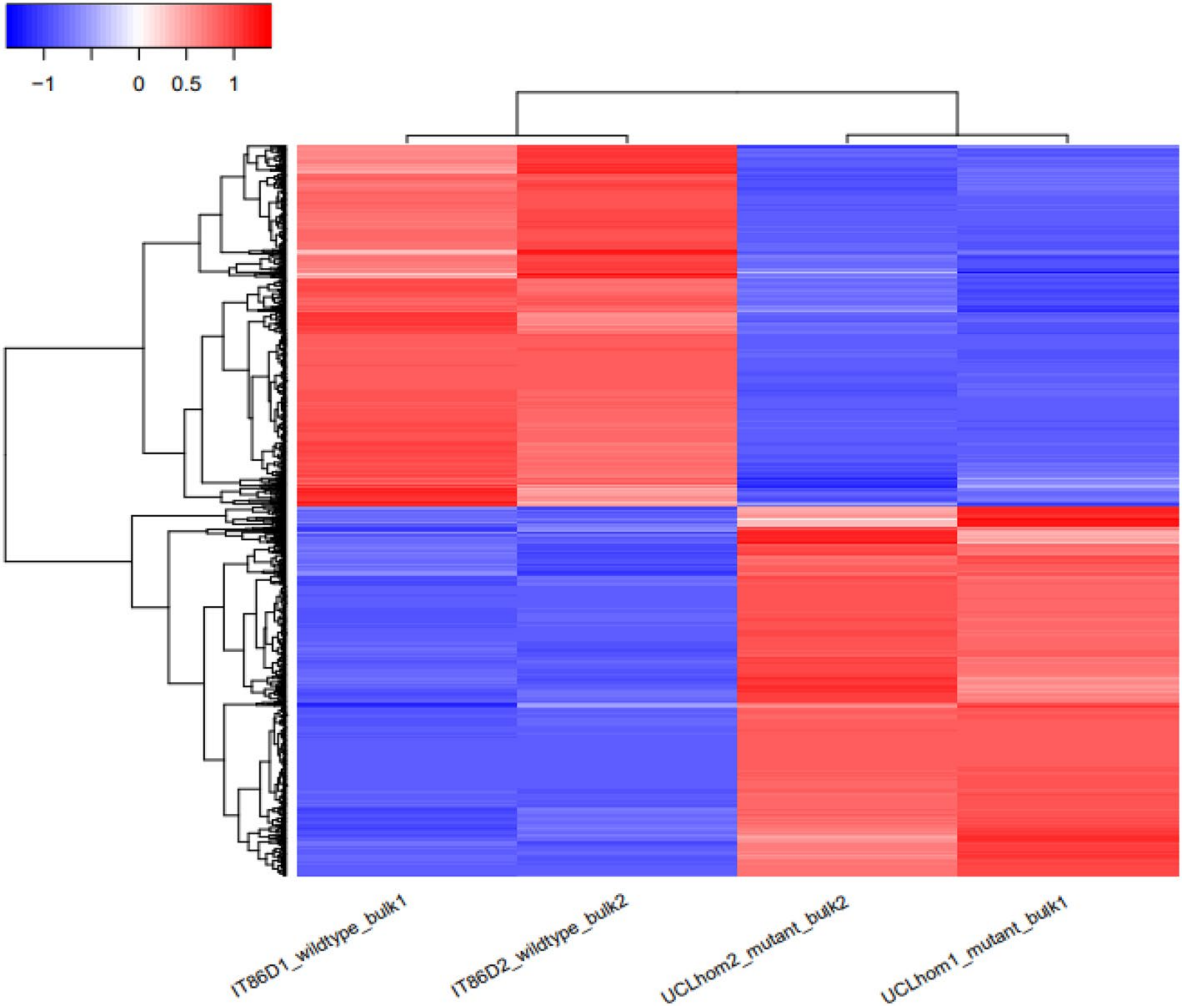
Heatmap of RNA-seq-based differential gene expression between wild-type (IT86D-101) and UCL homozygous mutant. UCL, unifoliate curled leaf; IT86D1_wildtype_bulk1, wild-type biological bulk replicate 1; IT86D2_wildtype_bulk2, wild-type biological bulk replicate 2; UCLHom1_mutant_bulk1, UCL homozygous mutant biological bulk replicate 1; UCLHom2_mutant_bulk2, UCL homozygous mutant biological bulk replicate 2.

Differential gene expression between the wild-type and UCL mutant was analyzed using gene counts obtained by implementing ***featureCounts***^35^ on the mapped collection of the datasets produced by *RNA STAR* mapper. With the aid of *DESeq2*^36^, we normalized the gene counts and identified 2,106 significantly differentially expressed genes (DEGs) in the UCL mutant genome (adj. p < 0.05 and abs(log_2_(FC)) > 1; abs, absolute; FC, fold change). Of the DEGs, 1,068 (~ 51%) are up-regulated and 1,038 (~ 49%) are down-regulated (Fig. 2; see also Table S3). To make sense of the large number of the DEGs, we sorted the genes from the most highly expressed to the least expressed in the wild-type, and applied *g:Profiler*, a leading functional enrichment web resource, to identify significantly enriched or depleted functional Gene Ontology (GO) terms^37,38^. A multi-query functional enrichment analysis was performed, with each query consisting of 50 genes. The results of the functional enrichment analysis showed 21 significantly enriched terms, and 10 significantly depleted terms (p (SCS) < 0.05; SCS, Set Counts and Sizes)^38^ (See Table S4 and S5), indicating a complex interplay of biological functions involving plastid, chloroplast, cell wall and extracellular region in the UCL mutant genome. The identified enriched or depleted functional GO terms will guide further research on compound leaf patterning and other functionally compromised mechanisms in the mutant.

### Compound leaf and UCL mutant phenotype-associated DEGs

**Figure 3:**
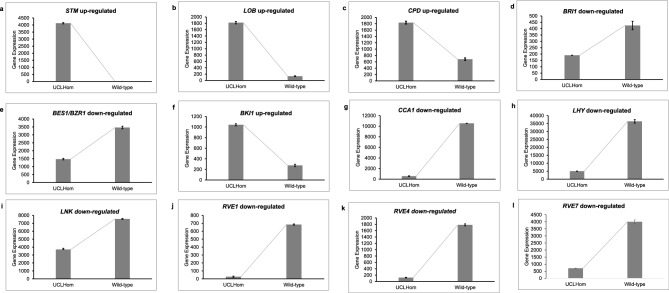
Differential expressions of gene candidates associated with UCL mutant phenotype. (**a)** Up-regulation of *VunSTM* in UCLHom; (**b)** Up-regulation of *VunLOB* in UCLHom; (**c)** Up-regulation of *VunCPD* in UCLHom; (**d)** Down-regulation of *BRI1* in UCLHom; **e.** Down-regulation of *VunBES1*/*BZR1* in UCLHom; (**f)** Up-regulation of *VunBKI1* in UCLHom; (**g)** Down-regulation of *VunCCA1* in UCLHom; (**h)** Down-regulation of *VunLHY* in UCLHom; (**i)** Down-regulation of *VunLNK* in UCLHom; (**j)** Down-regulation of *VunRVE1* in UCLHom; (**k)** Down-regulation of *VunRVE4* in UCLHom; (**l)** Down-regulation of *VunRVE7* in UCLHom; UCL, unifoliate curled leaf; UCLHom, UCL homozygous mutant.

Of the functionally characterized genes associated with compound leaf development^5^, we identified, among the DEGs in the UCL mutant, cowpea orthologs (best protein matches) of *SHOOT MERISTEMLESS* (*STM*; *Vigun06g157800*), *LATERAL ORGAN BOUNDARIES* (*LOB*; *Vigun02g150500*), *BRASSINOSTEROID INSENSITIVE 1* (*BRI1; Vigun02g046500*), and cytochrome P450 (CYP450) genes, including *CONSTITUTIVE PHOTOMORPHOGENIC DWARF* (*CPD*; *Vigun01g226500*) (Fig. 3a–d), which is highly expressed in leaves and strongly associated with *BRI1*-dependent BR signaling^39^.

The insignificant expression of cowpea *STM* (*VunSTM*) in the wild-type and its high (~ 4000-fold) expression in the UCL mutant (Fig. 3a) suggests that the expression of *HOMEOBOX*/*KNOX*-like genes in cowpea is suppressed under wild-type conditions, which is consistent with reports that indicated that *KNOX*-like genes are excluded from the leaves of some legumes^1,5^. *M. truncatula* ortholog of *LOB*, *ELONGATED PETIOLULE1* (*ELP1*)/*PETIOLULE-LIKE PULVINUS* (*PLP*), is involved in leaf movements, mediated by rhythmic swelling, and shrinking of the motor cells of pulvinus^40,41^. Altered expression of *ELP1*/*PLP* produces mutant plants with elongated pulvini, where the motor cells are being replaced by petiole-like epidermal cells^42^. As shown in Fig. 1b, the pulvini of the leaves of the UCL mutant are elongated and curled. This suggests that the up-regulation of the *VunLOB* (Fig. 3b) in the UCL mutant results in petiolule-like pulvini (Fig. 1b), comparable to the response of *M*. *truncatula* to ectopic expression of *ELP1*/*PLP*^40,42^.

Also, *LOB* negatively regulates BR accumulation^43^, hence the differential expressions of *CPD* and *BRI1* in the UCL mutant genome are particularly informative. *CPD*, known to be more actively transcribed than other BR-biosynthetic CYP450 genes, is one of the CYP450 genes that encode BRs biosynthesis rate-limiting enzymes^44–46^. Consequently, the up-regulation of *CPD* in the UCL mutant is likely associated with deficiency in BRs, as transcription of *CPD* is negatively controlled by BRs, and a *brassinosteroid insensitive 1* (*bri1*) mutant has been reported to have an up-regulated expression of *CPD* in *A. thaliana*^39,47,48^. In *A. thaliana*, *BRI1*, a leucine-rich repeat receptor kinase (LRR-RLK), initiates BR signaling cascade, and *BRI1 KINASE INHIBITOR1* (*BKI1*) prevents the signaling of *BRI1*^49,50^. Among other components of BRs signaling cascade, *BRI1-EMS SUPPRESSOR1* (*BES1*) and *BRASSINOZOLE-RESISTANT1* (*BZR1*) are positive regulators of *BRI*^51,52^. Intriguingly, we found that cowpea orthologs of *BRI1*, *BES1/BZR1* (*Vigun05g181100*) and *BKI1* (*Vigun09g239100*) are differentially expressed in the UCL mutant: *BRI1* and BES1/BZR1 are down-regulated, while *BKI1* is up-regulated (Fig. 3d–f). Based on the expressions of the BR signaling-associated genes in the UCL mutant, we contend that the up-regulation of *VunBKI1* in the UCL mutant negatively regulates the activity of *VunBRI1* and *VunBES1/BRZ1*, resulting in BR deficiency, indicated by the BR-deficient-like phenotypes observed in the mutant (Fig. 1a, b)^27,28^.

### Predicted expression and co-expression analyses associate CCA1, LHY, STM, CPD, RVE and LNK with leaf development

*CIRCADIAN CLOCK ASSOCIATED 1* (*CCA1*; *Vigun09g004100*) and *LATE ELONGATED HYPOCOTYL* (*LHY*; *Vigun10g153300*) MYB-related TFs are also highly down-regulated in UCLHom (Fig. 3g, h). To investigate the association of these MYB TFs with leaf development in cowpea, we conducted in silico gene expression analysis for the TFs and the DEGs associated with compound leaf development. As we are not aware of any cowpea electronic gene expression browser, we used soybean (non-IRLC legume) orthologs of cowpea *CCA1* (*Glyma.03g261800*), *LHY* (*Glyma.07G048500*), *STM* (*Glyma.15G111900*), and *CPD* (*Glyma.11G228900*) to predict the organs where the genes likely express during cowpea development. Using the Soybean electronic fluorescent pictograph (eFP) browser at http://bar.utoronto.ca^53,54^, under Developmental Map view, we found that *CCA1*, *LHY*, *STM*, and *CPD* show medium to strong expressions in SAM and leaves (See Figure S3), indicating their involvement in leaf development, especially *LHY* and *STM* which are strongly expressed in SAM. The expression of genes associated with compound leaf morphogenesis in SAM and young leaf primordia is required to maintain primary morphogenetic activity in compound leaf development^5,55^.

To identify other DEGs that are highly positively correlated with cowpea *CCA1* and *LHY* (r ≥ 0.7), we used *A*. *thaliana* orthologs of *CCA1* and *LHY*, *At5g02840* and *At1g01060*, respectively, to conduct co-expression analysis at http://bar.utoronto.ca/ExpressionAngler/^56^. From our results, apart from *CCA1* and *LHY* highly co-expressing with each other, a total of 41 other genes are highly co-expressed with either *CCA1* or *LHY* or both (See Table S7). More importantly, among all the co-expressed genes, only seven genes are differentially expressed (down-regulated) in the mutant (See emboldened gene IDs in Table S7), indicating that the other co-expressed genes are not involved in the UCL mutation. Among the seven co-expressed DEGs are *DENTIN SIALOPHOSPHOPROTEIN-LIKE PROTEIN* (*Vigun06g223900*), orthologous to *A. thaliana NIGHT LIGHT-INDUCIBLE AND CLOCK-REGULATED1* (*LNK1*) (Fig. 3i), and *PROTEIN REIVELLE4-RELATED* (*RVE4*; *Vigun07g078900*) (Fig. 3k), orthologous to *A. thaliana REVEILLE8* (*RVE8*). *LNK1* and *RV8* are transcriptional coactivators and positive regulators of the components of circadian rhythm central oscillator; *RVE8* specifically targets the evening element of *CCA1/LHY* promoters^57–59^. Considering the importance of *RVE* genes in circadian clock-regulated developmental processes in plants^59–62^, we further examined the expressions of other *VunRVE* gene homologs in the wild-type and UCL mutant genomes. We found that *VunRVE1* and *VunRVE7* are also down-regulated in the UCL mutant (Fig. 3j, l). This suggests that the components of the circadian clock central oscillator and their coactivators are repressed in the UCL mutant genome. Like *CCA1, LHY, STM and CPD*, which are strongly expressed in leaves and/or SAM, soybean orthologs of *VunRVE1* (*Glyma.10g048500*), *VunRVE4* (*Glyma.15g053000*), *VunRVE7* (*Glyma.18g044200*) and *VunLNK* (*Glyma.16g217700*) are strongly expressed in leaves and/or SAM (See Figure S3e – S3h), suggesting their association with leaf development.

### De novo transcriptome assembly and quantification

To ensure a comprehensive identification and quantification of all the transcripts necessary for a reliable analysis of alternative splicing, we applied the mapped sequences of the RNA-seq datasets to perform de novo transcriptome reconstruction. With the aid of ***StingTie***^63^, we assembled and quantified the transcripts from the mapped collection of the libraries, guided by *Vunguiculata_540_v1.0* reference genome and *Vunguiculata_540_v1.2* reference annotation. To generate a new non-redundant reference transcriptome annotation, the assembled transcripts were merged with *Vunguiculata_540_v1.2* reference annotation, using *StingTie-merge*^63^. We evaluated the annotation accuracy using *GFFCompare*^64^ to compare the merged reference annotation with *Vunguiculata_540_v1.2* reference annotation. From the accuracy statistics output of *GFFCompare* (See Note S1), at all feature levels, sensitivity ranged from 99.1% to 100%, while precision ranged from 88.7% to 97.7%, indicating high proportions of query features that agree with corresponding reference annotation features. Also, out of the 61,283 query mRNAs in 33,697 loci across all the input datasets, no exon or locus was missed. While only 1 intron out of 150,963 introns (~ 0%) was missed, 3,921 novel exons, 2007 novel introns, and 2,043 novel loci were identified (See Note S1). Apart from validating the annotation accuracy, the *GFFCompare* statistics show that, although gene/transcript databases are large and reasonably representative, a de novo transcriptome assembly has the power of revealing novel transcript structures which are not represented in databases. The validated reference transcriptome we generated from *StingTie-merge* enabled us to accurately quantify transcript-level expressions, which we used to conduct genome-wide analysis of alternative splicing (AS) and isoform switch (IS).

### Isoform switching underlies differential expressions of CCA1, LHY and CPD in the UCL mutant

To identify the predominant AS type resulting in significant functional switches from wild-type to mutant, the transcript-level gene expressions of all the transcripts in the RNA-seq datasets were analyzed using *IsoformSwitchAnalyzeR*^65^. We generated a raw switch list and their corresponding nucleotide and amino acid sequences, and used the amino acid and nucleotide sequences to, respectively, identify coding domains, and predict the coding potential of the transcripts, using *CPAT*^66^. With the switch list, protein domains and coding potential data, genome-wide AS and their functional consequences in the mutant were analyzed. The genome-wide gene expression plots indicate that there are genes in the mutant genome that are both differentially expressed (large log_2_FC) and contain isoform switches (red color) (See Figure S3a)^65^. The results of comparing the number of isoforms significantly differentially used between the wild-type and mutant genomes resulting in at least one splice event indicate that there is statistically significant alternative transcription termination sites (ATTS) events in the mutant genome (See Figure S3b)^65^. Also, consequence enrichment analysis shows that there is a significant gain in nonsense-mediated mRNA decay (NMD) insensitivity in the mutant genome (See Figure S3c). This implies a likely accumulation of truncated proteins translated from NMD-insensitive transcripts in the mutant^67–69^. NMD, a conserved mRNA quality control mechanism in eukaryotes, identifies and destroys aberrant mRNA containing premature termination codons (PTCs), and regulates the expression of normal transcripts^67,68,70–73^.

**Figure 4:**
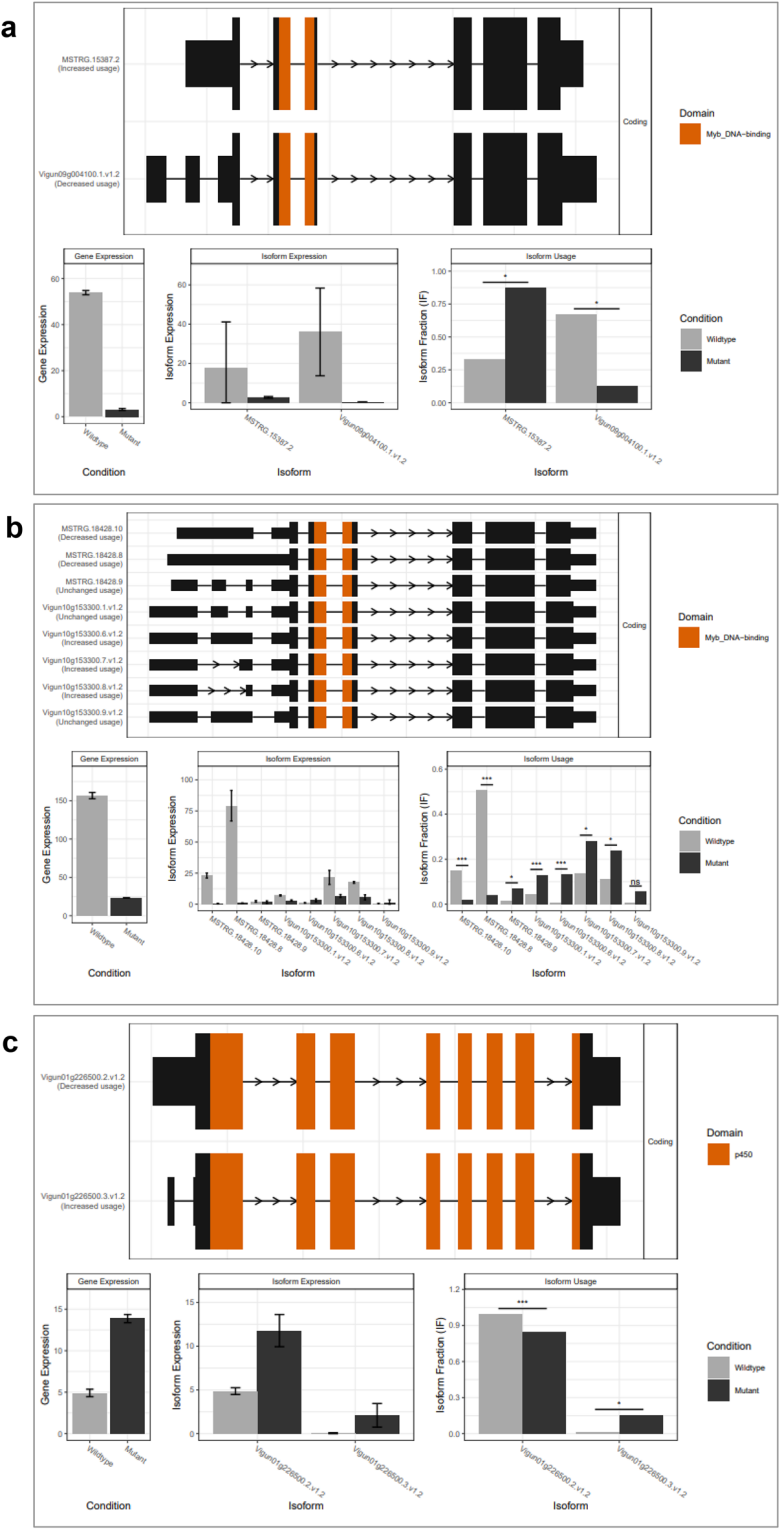
Isoform switch-dependent differential expressions of genes associated with UCL mutant. (**a)** Down-regulation of *VunCCA1* in UCLHom mutant resulting from decreased expression and usage of the wild-type isoform; (**b)** Down-regulation of *VunLHY* in UCLHom mutant resulting from decreased expressions and usage of wild-type isoforms; (**c)** Up-regulation of *VunCPD* resulting from higher expression and usage of the mutant isoform; UCL, unifoliate curled leaf; UCLHom, UCL homozygous mutant.

From the genome-wide AS analysis, we discovered 286 genes with statistically significant switches resulting in predicted functional consequences (q < 0.05) in the mutant genome, out of which 32 genes are significantly differentially expressed (See Table S6. The 32 IS-dependent DEGs are among the 2,106 DEGs identified using the reference-based approach of differential gene expression analysis, validating the two approaches of differential gene expression analysis we adopted. The two IS-dependent most down-regulated genes are *CIRCADIAN CLOCK ASSOCIATED 1* (*CCA1*; *Vigun09g004100*) and *LATE ELONGATED HYPOCOTYL* (*LHY*; *Vigun10g153300*) MYB-related TFs (Fig. 4a and b), whose orthologs in *A. thaliana* are key components of circadian rhythm central oscillator^74–77^. Interestingly, we found that the up-regulation of *CPD* in the UCL mutant is also IS-dependent (Fig. 4c), which suggests a possible cross-talk between the expressions of the isoforms of *CCA1* or *LHY* or both and *CPD* in cowpea.

### Genomic variants in the UCL mutant candidate genes

**Figure 5:**
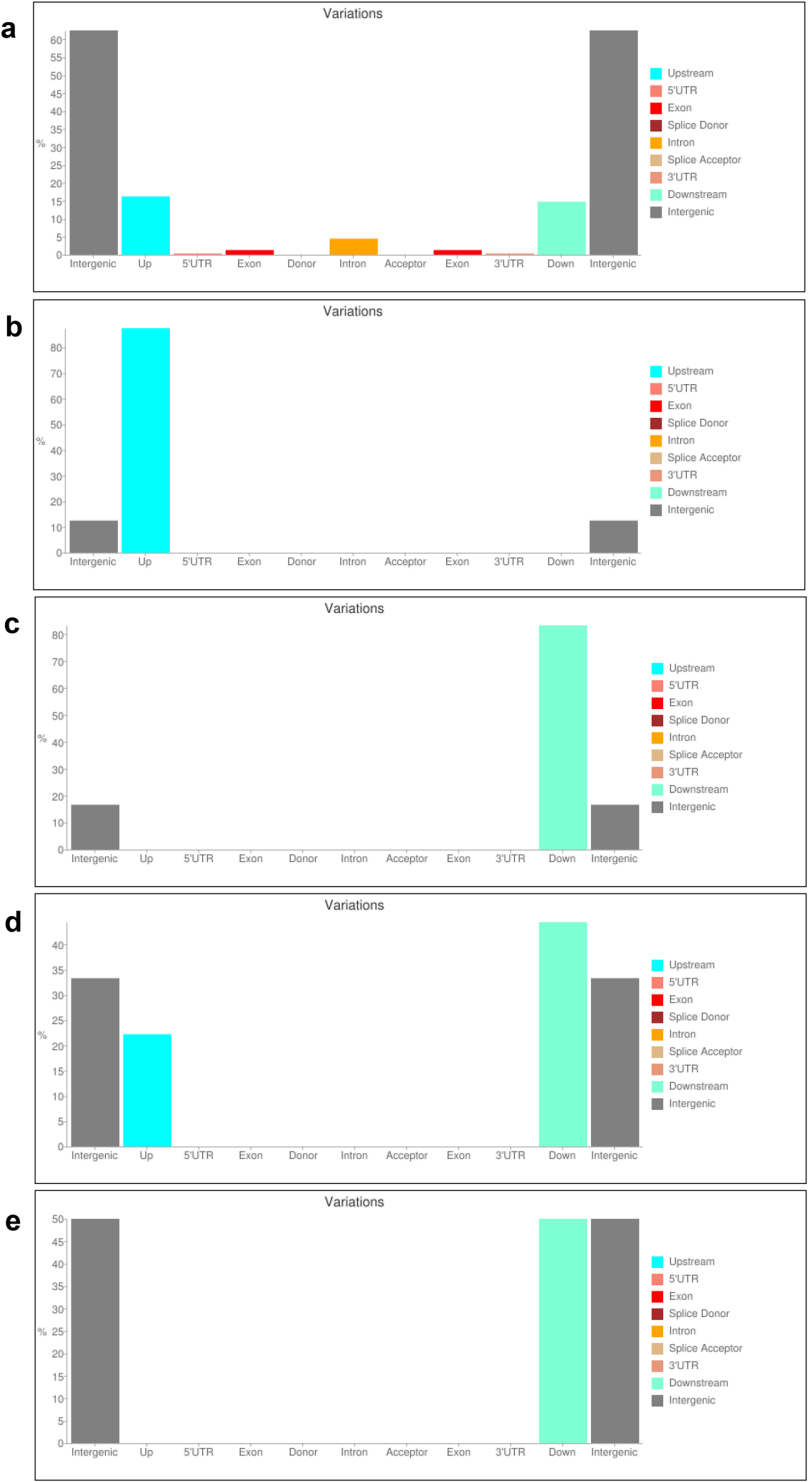
Regions of genomic variants in UCL mutant. **(a)** Total genomic variants; (**b)** *VunLHY* variants; (**c)** *VunRVE4* variants; (**d)** *VunBRI1* variants; (**e).** *VunLOB* variants; UCL, unifoliate curled leaf.

We applied *FreeBayes*, a reputable Bayesian genetic variant detector^78^, to jointly call a total of 7,700,700 variant sites from the UCL mutant and revertant mapped genome sequences. To identify UCL genome-specific variants, *MiMohD VCF Filter*^79^ was applied to extract 69,449 homozygous variant sites spread across all the chromosomes in the UCL mutant genome corresponding to homozygous wild-type loci in the revertant genome. This filtering ensured that recombinant variant sites which are common to UCL mutant and revertant genomes were excluded from the UCL variants list. The filtered variants were annotated using *snpEff eff*^80^, and reported with *MiMohD Report Variants*^79^. The variants are genome-wide and are mostly in the intergenic, upstream, and downstream regions (Fig. 5a), making the UCL mutant a potential source of genes for cowpea breeding. Of the 12 UCL phenotype-associated DEGs in the UCL mutant genome (Fig. 2), only *LHY*, *RVE4*, *BRI1* and *LOB* have either upstream or downstream nucleotide variations in the UCL mutant genome (Fig. 5b–e; See also Table S8 – 11). *LHY* is affected by two upstream substitutions, TCG/CTA (chrVu10: 37,344,133) and CA/TC (chrVu10: 37,344,604) (See Table S8); a downstream TATATATGTA insertion (chrVu07: 10,891,928) affects *RVE4* (See Table S9); three downstream substitutions, A/T (chrVu02: 18,476,009), C/T (chrVu02: 18,476,011) and ACA/GCC (chrVu02: 18,483,367), affect *BRI1* (See Table S10), and *LOB* is affected by two downstream substitutions G/T (chrVu02: 29,738,970) and A/T (chrVu02: 29,738,992) (See Table S11). *LHY* and *RVE4* co-express with *CCA1* (See Table S7); *LNK*, *RVE1* and *RVE7* are positive regulators of *CCA1*/*LHY*^57–59^, and *BRI1*, *CPD*, *BES1*/*BZR1*, *BKI1* and *LOB* are all associated with BR biosynthesis and accumulation^43,49,50^. The functional relationships among the gene candidates suggest that the differential expressions of the candidates without genomic variants in the UCL mutant are functional responses to expressions of *LHY*, *RVE4*, *BRI1* and *LOB*.

## Discussion

### IS-dependent repression of circadian rhythm-regulated genes control leaf patterning in cowpea

For optimal utilization and allocation of resources, plants rely on endogenous circadian rhythms to synchronize internal physiological processes with diurnal cycles and seasonal environmental changes^76,81,82^. About 6% of gene expression patterns, and various developmental processes in plants exhibit circadian rhythms^83,84^. In *A. thaliana*, *CCA1*, *LHY* and *TOC1* are components of the central oscillator of circadian clock; *CCA1* and *LHY*, which are morning expressed genes^85,86^, are needed for the maintenance of circadian rhythm under constant light, and *TOC1*, an evening expressed gene^87,88^, expresses earlier than expected, when *CCA1* and *LHY* are deactivated^75^. Disruptions in the expressions of *CCA1* and *LHY* affect the period of circadian rhythms in gene expression and leaf movements^57,58,75,85,89,90^. *RVE* genes and their transcriptional coactivators, *LNK1 and LNK2*, are positive regulators of the components of central oscillator of circadian clock^59–61,76^. The roles of *CCA1*, *LHY*, *TOC1* and other circadian rhythm-associated genes have also been studied in various legume species^91^. In cowpea, *VunTOC1*, *VunLHY*, *VunELF3* and *VunGI* are associated with circadian clock function in seed filling and leaves^92^. Our results have shown that *VunCCA1*, *VunLHY*, *VunLNK, VunRVE1, VunRVE4* and *VunRVE7* are all down-regulated in the UCL mutant, suggesting a disruption in the mutant’s circadian clock central oscillator. While the down-regulation of *VunCCA1* and *VunLHY* are IS-dependent, the repression of *VunLNK* and the three *VunRVE* genes are not associated with IS. Of all the circadian rhythm-regulated DEGs associated with leaf development, only *VunLHY* and one of its positive regulators, *VunRVE4*, have mutations in their upstream and downstream regions, respectively. This suggests that the repressed expressions of the other functionally related candidates are likely dependent on the repression of *VunLHY* or *VunRVE4* or both. We, therefore, hypothesize that the genomic variants in *VunLHY* or *VunRVE4* or both resulted in decreased expressions of the wild-type isoforms of *VunLHY* and *VunCCA1* in the UCL mutant, compelling the compensatory usage of the alternative isoforms that resulted in the development of unifoliate leaves in the mutant. Put differently, optimal expressions of the wild-type isoforms of *VunLHY* and *VunCCA1*, and their positive regulators, *VunLNK*, *VunRVE1*, *VunRVE4* and *VunRVE7*, are required for compound leaf development in cowpea. Previous studies have associated *S. lycopersicum* and *M. truncatula* MYB TFs with compound leaf patterning^55,93–96^. Increased expression of the tomato MYB TF, *Trifoliate* (*Tf*), in young leaf primordia sustains morphogenetic activity, resulting in a surge in leaf dissection and inhibition of cell differentiation^55^. The tomato *CLAU* gene, another MYB TF, influences tomato leaf morphogenesis in a dose-dependent manner. Loss of function mutation (*clau*) causes extended morphogenesis and elaborate leaves, but over-expression of *CLAU* results in simplified leaves with primary leaflets^55,97,98^. Mutant of *M. truncatula PHANTASTICA* (*MtPHAN*), another MYB TF, is reported to exhibit leaf curling, deep serration of leaf margins and shortened petioles^96^. The association of IS-dependent repression of *VunCCA1*/*VunLHY* in the switch from compound leaves in the wild-type to unifoliate leaves in the UCL mutant is yet another evidence of the role of alternative splicing in regulating the functions of genes^69,99,100^. Although MYP TFs play diverse roles in plants^101–104^, our discovery of the likely implication of IS-dependent differential expression of *VunCCA1*/*VunLHY* in compound leaf development of a legume species is novel.

The insignificant expression of *VunSTM*, a KNOX gene, in the wild-type and its more than 4000-fold up-regulation in the UCL mutant, suggests that, under wild-type conditions, normal expressions of *VunCCA1*, *VunLHY*, *VunLNK*, *VunRVE1*, *VunRVE4* and *VunRVE7* either negatively regulate *STM* expression or replace its functions in cowpea primary morphogenesis, consistent with reports which have shown that KNOX genes do not express in the leaves of some legume species^1,5^.

### Down-regulation of BRI1 results in BR-deficient-like phenotype in the UCL mutant

BRs are phytohormones which regulate various processes in plants, and CYP450 genes, notably *CPD* and *CYP85A2*, encode enzymes responsible for the biosynthesis of BRs^42,46^. *CPD* expression is predominantly regulated by the *BRI1*-dependent BR signaling pathway; *bri1* mutant has an elevated expression of *CPD*, and the over-expression of *BKI1*, a *BRI1* negative regulator, up-regulates *CPD* expression^39,47,49^. *BRI1*initiates the BR signaling cascade^42^, and *BKI1* regulates plant architecture by negatively regulating *BRI1* in the BR pathway^49,105^. Our differential gene expression analysis shows that *BKI1* and *CPD* are up-regulated in the UCL mutant, while *BRI1* and its positive regulator, *BES1/BZR1*, are down-regulated, producing the BR-deficient-like phenotypes, especially the curled dark green leaves and reduced plant size observed in the UCL mutant^27,28^. The differential expressions of these functionally related BR signaling pathway genes suggest two hypotheses. First, the identified *BRI1* downstream variants in the UCL mutant compromise *BRI1*-dependent BR signaling pathway, resulting in the up-regulated expressions of *CPD* and *BKI1*. Second, repressed expressions of the components of the *BRI1*-dependent BR signaling is the output of the suppressed circadian clock endogenous oscillator of the UCL mutant^76^. The association of *CPD* with monooxygenase activity, iron ion binding, heme binding, and regulation of circadian rhythm^46,106–109^, some of the functionally enriched terms in the UCL mutant, strongly indicate that up-regulation of the *VunCPD*, and the corresponding down-regulation of *BRI1* in the UCL mutant have roles in producing the mutant phenotype. Also, down-regulation of *VunBRI1* is likely responsible for the functional depletion of response to red or far-red light and blue light signaling pathway in the UCL mutant^49,51,110^.

Taken together, our study has revealed important candidate genes, genomic variants and GO terms associated with the UCL mutant, laying a formidable foundation for future research that would aid in-depth understanding of the genetics of compound leaf development in cowpea. As our results suggest the involvement of various genes in the UCL mutant phenotype, future follow-up reverse genetic elucidation of the specific roles of the candidate genes, especially the candidates with genomic variants (*VunLHY*, *VunRVE4*, *VunBRI1* and *VunLOB*), will provide useful insights into the molecular mechanisms underlying leaf morphogenesis in cowpea. Based on our results, we have stated three hypotheses as directions for future studies to build on our work. We strongly recommend the inclusion of de novo genome-wide IS analysis in forward genetic screens targeted at identifying genes or gene candidates responsible for spontaneous aberrant phenotypes in plants.

## Methods

### Plant materials and growth chamber conditions

We worked with three wild-type cowpea cultivars, IT86D-1010, IT97K-499–35 and Sasaque, reported in our speed breeding protocol^111^, and a spontaneous mutant segregant from a self-pollinated progeny of IT86D-1010, which we have named UCL mutant. As previously reported^111^, Sasaque, a Japanese cowpea cultivar, was originally obtained from TJ Higgins of Commonwealth Scientific and Industrial Research Organization, Australia; IT86D-1010 and IT97K-499–35 are cowpea breeding lines produced by the International Institute of Tropical Agriculture (IITA), Nigeria, and made available to us through the Hy-Gain for Smallholders Project (https://hy-gain.org/). Seeds of all the genotypes, including the UCL mutant, are maintained in the cowpea gene bank at ALRC, Tottori University, Japan. All the experimental plants were grown under the same controlled environmental conditions: 10 h photoperiod, 23 °C night/25 °C day temperature, and 70% day/75% night relative humidity.

### Agro-morphological and genetic characterization of the UCL mutant

Agro-morphological variation between IT86D-1010, UCLHet and UCLHom was studied under the same growth conditions, with the genotypes laid out in a completely randomized design with three replications. Each replication consisted of three plants/genotype, and data were taken on total fresh shoot biomass, days to 50% flowering, pod length, number of pods/plant, number of seeds/pod and 100 seed weight. The mean and median of the data for each measured trait were either equal or nearly equal, indicating normality of the data. Using SPSS Statistics software (version 29.0), the data were subjected to analysis of variance, and significantly different means (p < 0.01) were separated using the Least Significant Difference Test. To determine whether the mutant locus is dominant or recessive, we observed segregation in both self- and cross-pollinated generations in our speed breeding facility. In the first self-pollinated generation, we observed 24 segregants harvested from the original mutant plant identified in the field. From the 24 segregants, we bulk-harvested seeds from UCLHet, UCLHom and revertant segregants. Using the UCLHet bulk, we cultivated and observed 200 plants in F_2_, and continued the same process until F_6_. With 50 seeds per generation, we also observed self-pollinated generations of UCLHom and revertant up to F_6_ for each of the genotypes. To observe allelic segregation in cross-pollinated populations, we made reciprocal crosses between the mutant genotypes (UCLHom and UCLHet) and the three wild-type cowpea cultivars mentioned under plant materials. This afforded us the opportunity of observing the transmission of the mutant locus in different genetic backgrounds.

### RNA purification and sequencing

We extracted total RNA from frozen young leaves sampled from two weeks old seedlings of IT86D-1010 and confirmed self-reproducing UCLHom plants, using MagMAX Plant RNA Isolation Kit (Applied Biosystems). RNA quality was determined by NanoDrop 2000C spectrophotometer (Thermo Scientific), and quantification was done with Qubit 2.0 Fluorometer (Invitrogen). For each genotype, two bulked biological replicates, composed of RNA samples from 20 plants, were used for preparation of sequencing libraries. Strand-specific paired-end poly(A)-selected RNA-seq libraries of the bulked biological replicates prepared using TruSeq Stranded mRNA Library Prep Kit (Illumina) were sequenced with DNBSEQ-G400 (MGISEQ-2000RS) short reads sequencer.

### RNA-seq data analysis

Before downstream applications, initial read quality assessment was done with FastQC (https://www.bioinformatics.babraham.ac.uk/projects/fastqc/), after which the sequences were processed by *fastp*^29^. *RNA STAR* mapper^30^ was used to align the *fastp*-trimmed sequences to *Vunguiculata_540_v1.0* reference genome, with *Vunguiculata_540_v1.2* reference annotation as gene model (https://data.jgi.doe.gov/refine-download/phytozome?genome_id=540). We conducted a multi-sample 2-pass mapping, which enabled us to accurately discover and quantify splice junctions^31^. To analyze differential gene expression between the wild-type and UCL mutant, we applied *featureCounts*^35^ on the mapped collection of the datasets produced by *RNA STAR* mapper to obtain gene counts, after which *DESeq2*^36^, was applied to conduct differential gene expression between the datasets. GO analysis of the DEGs was conducted with *g:Profiler*, a leading functional enrichment web resource^37,38^.

With the aid of *StingTie*^63^, we assembled and quantified the transcripts from the mapped collection of the libraries, guided by *Vunguiculata_540_v1.0* reference genome and *Vunguiculata_540_v1.2* reference annotation. The assembled transcripts were merged with *Vunguiculata_540_v1.2* reference annotation to generate a novel non-redundant reference transcriptome annotation. Annotation accuracy was evaluated using *GFFCompare*^64^ to compare the merged reference annotation with *Vunguiculata_540_v1.2* reference annotation. With the aid of *IsoformSwitchAnalyzeR*^65^, genome-wide AS was analyzed using the transcript-level gene expressions of all the transcripts in the datasets. We generated a raw switch list and their corresponding nucleotide and amino acid sequences, and used the amino acid and nucleotide sequences to, respectively, identify coding domains, and predict the coding potential of the of transcripts, using *CPAT*^66^. For all the RNA-seq data analyses reported here, we used RNA-seq data analysis tools either in the Europe (https://usegalaxy.eu/welcome/new) or USA (https://usegalaxy.org/) instance of Galaxy.

### Prediction of expression and co-expression of candidate genes

Electronic gene browser at http://bar.utoronto.ca^53,54^ was applied to predict the developmental expression patterns of the gene candidates, and co-expression analysis was performed with *ExpressionAngler* at http://bar.utoronto.ca/ExpressionAngler/^56^.

### Genome variant analysis

We studied the variants in the UCL mutant genome using shotgun sequences of purified bulked DNA samples of F_2_ segregants of IT97K-499–35 × UCLHom hybrid. NucleoMag Plant Genomic DNA Extraction Kit (Takara Bio USA) was used to extract and purify DNA samples from 10 seedlings each of UCLHet and UCLHom, and 20 seedlings of wild-type revertant. Combined DNA samples of UCLHet and UCLHom segregants constituted the UCL mutant bulk, and the DNA samples of the 20 wild-type revertant plants were bulked to create the revertant bulk. The two libraries, prepared using MGIEasy FS DNA Library Prep Set, were sequenced with DNBSEQ-G400 short reads sequencer. The sequence reads were processed with *fastp*^29^, after which the *fastp*-passed sequences were mapped to *Vunguiculata_540_v1.0* reference genome, using *Bowtie2*^34^. *FreeBayes*^78^ was applied to jointly call variant sites from the UCL mutant and revertant mapped genome sequences, and *MiMohD VCF Filter*^79^ was used to extract homozygous variant sites in the UCL mutant genome corresponding to homozygous wild-type loci in the revertant genome. The filtered variants were annotated using *snpEff eff* with a custom *snpEff* database built from *Vunguiculata_540_v1.2* reference annotation, using *snpEff build*^80^. The variants were reported with *MiMohD Report Variants*^79^. All genomic data analyses reported here were conducted using genomic data analysis tools in the Europe instance of Galaxy (https://usegalaxy.eu/welcome/new).

## Ethics declarations

As stated under the plant materials section, Sasaque was obtained from TJ Higgins of Commonwealth Scientific and Industrial Research Organization, Australia; IT86D-1010 and IT97K-499–35 were provided by IITA, Nigeria. The authors identified the original UCL mutant plant as a spontaneous mutant segregant from the self-pollinated progeny of IT86D-1010 in the 2021 cowpea field cultivation at ALRC. To confirm reproducibility and identity of the mutant, the seeds harvested from the original mutant plant were cultivated under controlled growth chamber conditions, and the self- and cross-pollinated populations were observed over generations. Seeds of all the genotypes reported here, including the UCL mutant, are maintained in the cowpea gene bank at ALRC, Tottori University, Japan, a subsidiary of the cowpea gene bank of Japan’s National Agriculture and Food Research Organization (https://www.gene.affrc.go.jp/index_en.php). The seeds and plants of the mutant are currently used for research purposes only, and handling and all the research activities are done in accordance with all relevant guidelines.

### Supplementary Information

Supplementary Information 1.

Supplementary Information 2.

Supplementary Information 3.

## Data Availability

All associated data and information, including short reads of RNA-seq and whole genome shotgun sequence data of the analyzed genotypes, not included in the main article are published as supplemental information linked with the main article.
