# Supplementary material for: Genomic analysis of a spontaneous unifoliate mutant reveals gene candidates associated with compound leaf development in Vigna unguiculata [L] Walp
Source: Sci Rep. 2024 May 9;14:10654. doi: 10.1038/s41598-024-61062-x (PMC11082238; doi:10.1038/s41598-024-61062-x)
Supplement: Supplementary file 1 — Supplementary Information 1. [file 41598_2024_61062_MOESM1_ESM.pdf]

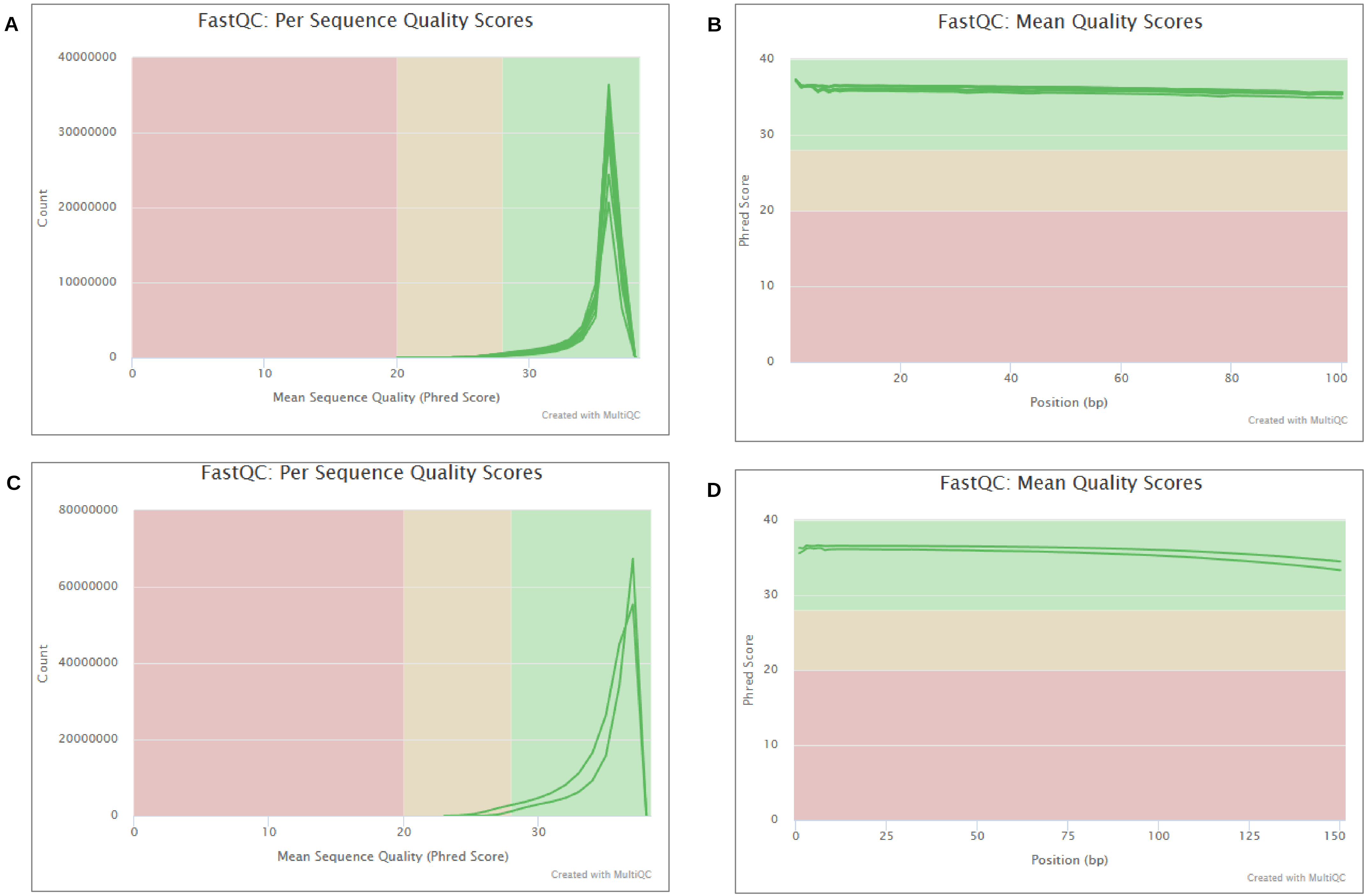

Figure S1 Sequence quality scores of RNA-seq and whole genome shotgun sequence datasets of IT86D-1010 and UCL mutant. A and B. Quality scores of RNA-seq datasets; C and D. Quality scores of and whole genome shotgun sequence datasets

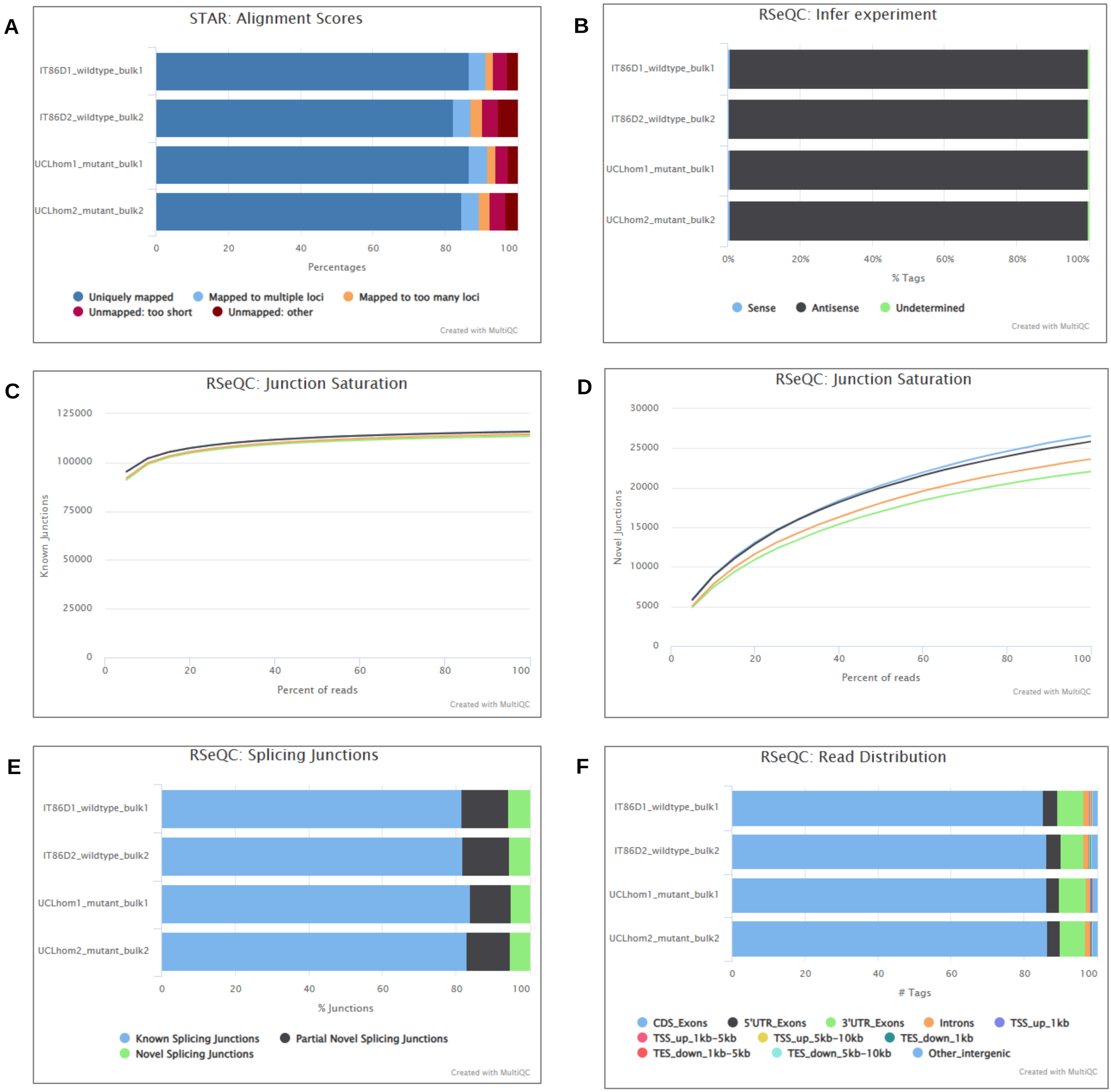

Figure S2 Post-alignment RNA-seq-specific quality metrics of RNA-seq datasets of IT86D-1010 and UCL mutant

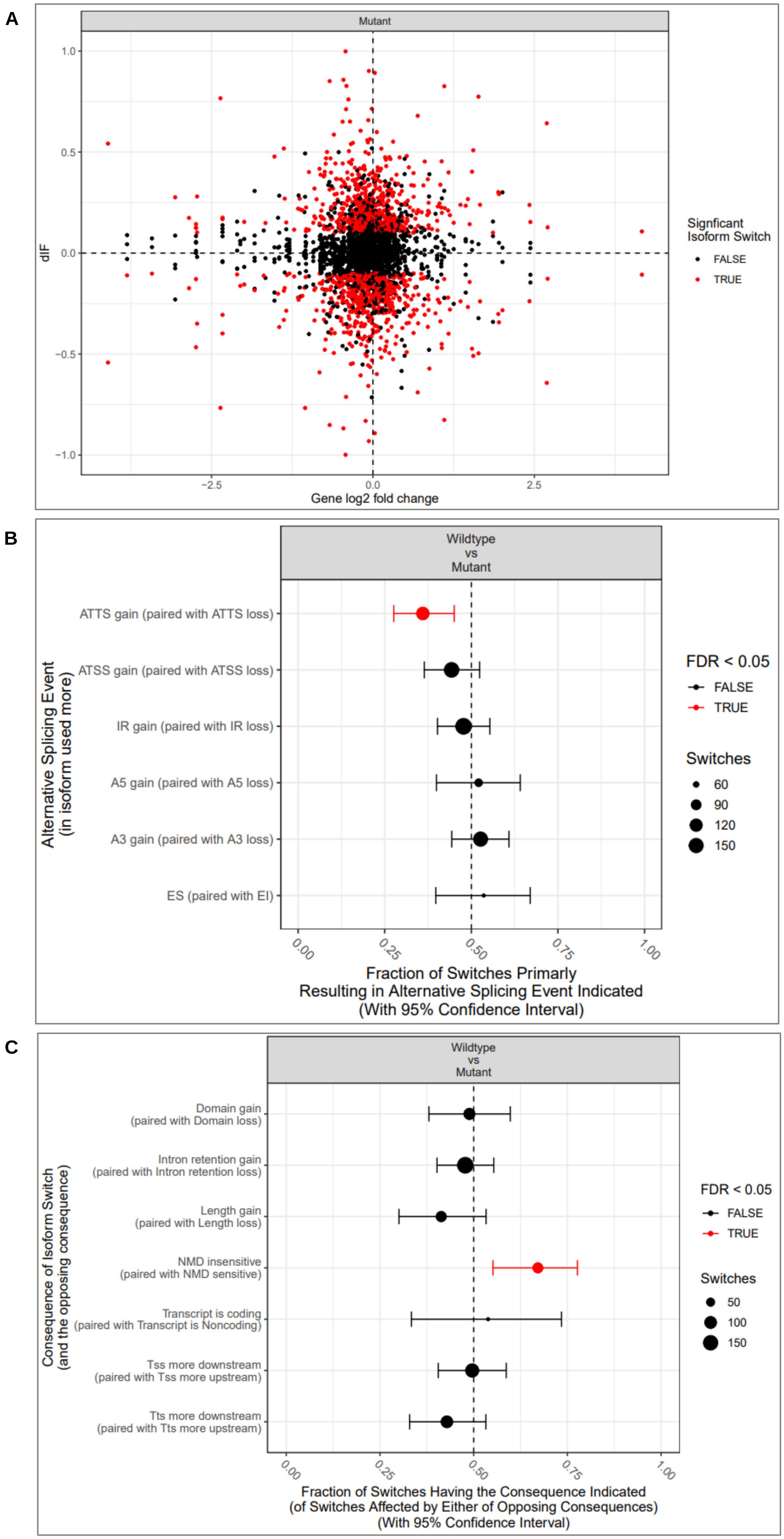

Figure S3 Genome-wide plots of alternative splicing and isoform switch from wild-type (IT86D-1010) to mutant (UCL)

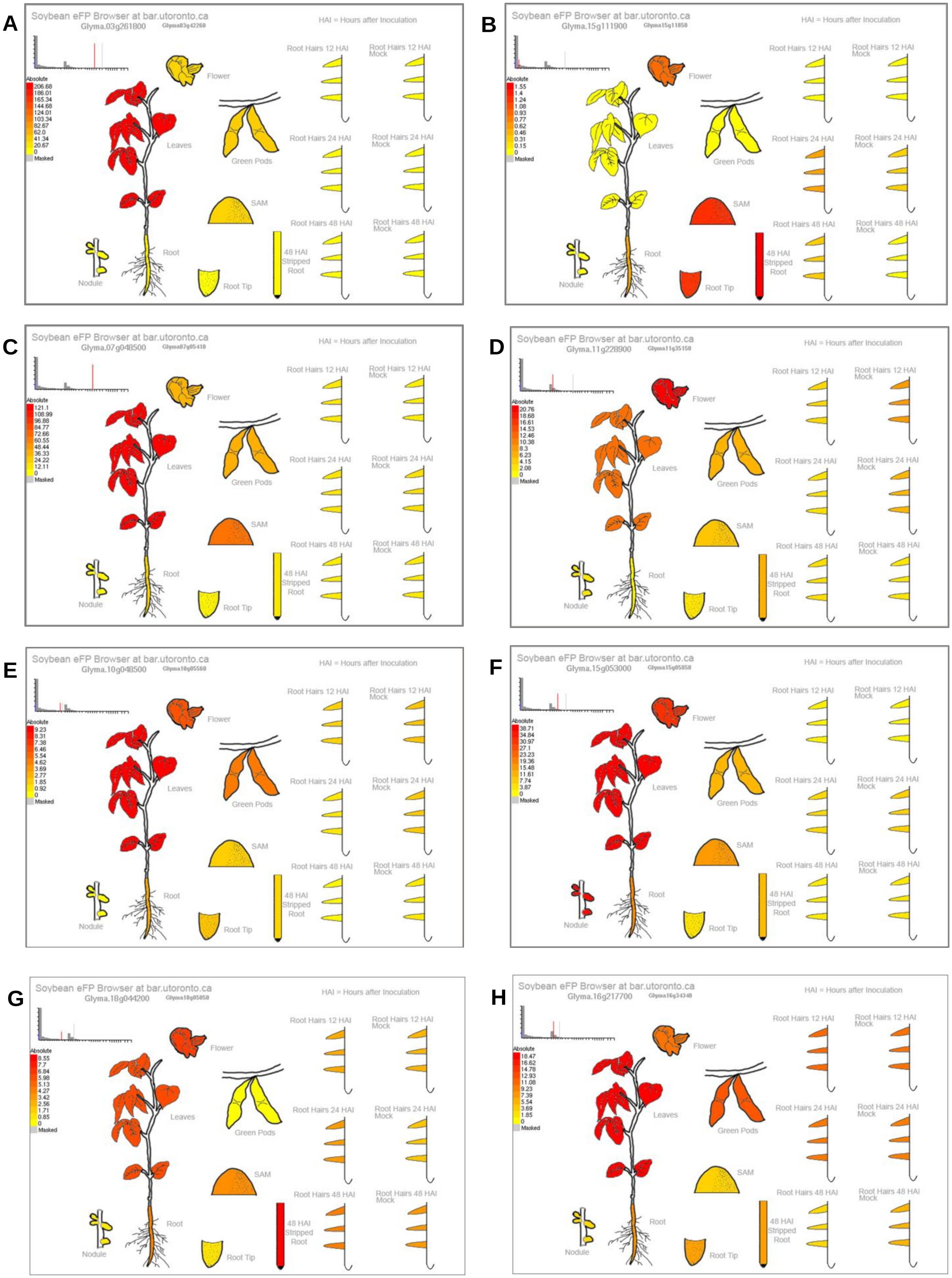

Figure S4 Predicted expressions of some gene candidates associated with UCL mutant. A. VunCCA1, B. VunSTM, C. LHY, D. VunCPD, E. *VunRVE1*, F. *VunRVE4*, G. *VunRVE7*, H. *VunLNK*

Table S1 UCL mutant locus segregation in F_1 hybrid progenies

Cross	Progeny size	No. of mutant (heterozygote)	No. of revertant
86D × UCLHet	100	57	43
UCLHet × 86D	100	52	48
86D × UCLHom	100	100	0
UCLHom × 86D	100	100	0
SAS × UCLHet	100	49	51
UCLHet × SAS	100	47	53
SAS × UCLHom	100	100	0
UCLHom × SAS	100	100	0
97K × UCLHet	100	54	46
UCLHet × 97K	100	52	48
97K × UCLHom	100	100	0
UCLHom × 97K	100	100	0

86D, IT86D-1010; 97K, IT97K-499-35; SAS, Sasaque; UCLHet, heterozygous unifoliate curled leaf; UCLHom, homozygous unifoliate curled leaf

Table S2 Summarized results of fastp trimming of RNA and genomic sequences

Dataset ID	RNA-seq datasets			
	Before filtering		After filtering	
	Total reads (M)	Total bases (G)	Total reads passed (M)	Total bases passed (G)
IT86D-1010_1	126.287418	12.628742	124.253380	12.370326
IT86D-1010_2	133.321948	13.332195	131.310080	13.077145
UCLHom_1	109.087334	10.908733	107.540108	10.702355
UCLHom_2	120.263786	12.026379	118.305644	11.772318
**Genome sequence datasets**				
Revertant bulk	295.170088	44.275513	295.169898	44.134702
UCL bulk	365.755054	54.863258	365.754824	54.699494

IT86D-1010_1, IT86D1_wild-type_bulk1; IT86D-1010_2, IT86D2_wild-type_bulk2; UCL, unifoliate curled leaf; UCLHom_1, UCLHom1_mutant_bulk1; UCLHom_2, UCLHom2_mutant_bulk2; average read length of RNA-seq datasets, 100 b; average read length of RNA-seq datasets, 150 b

Table S4 Functionally enriched GO terms in UCL mutant

Source	Term ID	Term name
GO:MF	GO:0016630	Protochlorophyllide reductase activity
GO:MF	GO:0004497	Monooxygenase activity
GO:MF	GO:0016705	Oxidoreductase activity, acting on paired donors, with incorporation or reduction of molecular oxygen
GO:MF	GO:0005506	Iron ion binding
GO:MF	GO:0000977	RNA polymerase II transcription regulatory region sequence-specific DNA binding
GO:MF	GO:0016634	Oxidoreductase activity, acting on the CH-CH group of donors, oxygen as acceptor
GO:MF	GO:0004185	Serine-type carboxypeptidase activity
GO:MF	GO:0004806	Triglyceride lipase activity
GO:MF	GO:0020037	Heme binding
GO:MF	GO:0005471	ATP:ADP antiporter activity
GO:MF	GO:0004568	Chitinase activity
GO:BP	GO:1901607	Alpha-amino acid biosynthetic process
GO:BP	GO:0009643	Photosynthetic acclimation
GO:BP	GO:0009228	Thiamine biosynthetic process
GO:BP	GO:0042546	Cell wall biogenesis
GO:BP	GO:0048588	Developmental cell growth
GO:BP	GO:0042752	Regulation of circadian rhythm
GO:CC	GO:0009536	Plastid
GO:CC	GO:0009507	Chloroplast
GO:CC	GO:0005618	Cell wall
GO:CC	GO:0005576	Extracellular region

GO, gene ontology; MF, molecular function; BP, biological process; CC, cellular component

Table S5 Functionally depleted GO terms in UCL mutant

Source	Term ID	Term name
GO:MF	GO:0140575	Transmembrane monodehydroascorbate reductase activity
GO:MF	GO:0004385	Guanylate kinase activity
GO:MF	GO:0016717	Oxidoreductase activity, acting on paired donors, with oxidation of a pair of donors resulting in the reduction of molecular oxygen to two molecules of water
GO:MF	GO:0004144	Diacylglycerol O-acyltransferase activity
GO:BP	GO:0006629	Lipid metabolic process
GO:BP	GO:0009135	Purine nucleoside diphosphate metabolic process
GO:BP	GO:0046037	GMP metabolic process
GO:BP	GO:0009639	Response to red or far-red light
GO:BP	GO:0009785	Blue light signaling pathway

GO, gene ontology; MF, molecular function; BP, biological process

Table S7 Highly co-expressed genes with *VunCCA1* and *VunLHY*

VunCCA1			***VunLHY***		
V. unguiculata	***A. thaliana* ortholog**	**r-value**	***V. unguiculata***	***A. thaliana* ortholog**	**r-value**
Vigun01g030600	At3g54500	0.866	Vigun01g030600	At3g54500	0.864
Vigun10g153300	At2g46830	0.834	**Vigun07g078900**	At3g09600	0.827
**Vigun07g078900**	At3g09600	0.832	Vigun09g004100	At5g02840	0.825
Vigun01g069600	At3g27090	0.806	Vigun07g020000	At5g06530	0.796
Vigun03g310000	At4g11960	0.803	**Vigun05g174200**	At1g78510	0.784
Vigun02g117700	At4g35780	0.785	Vigun03g310000	At4g11960	0.775
Vigun07g020000	At5g06530	0.772	**Vigun06g223900**	At5g64170	0.775
Vigun05g088900	At3g51730	0.767	Vigun09g250600	At2g31380	0.773
Vigun06g053400	At4g26860	0.726	Vigun04g068400	At3g02380	0.767
**Vigun09g153100**	At5g24120	0.725	Vigun02g117700	At4g35780	0.763
**Vigun06g223900**	At5g64170	0.723	**Vigun09g153100**	At5g24120	0.755
Vigun05g172300	At1g17080	0.722	Vigun03g183800	At4g33950	0.751
**Vigun03g001800**	At5g37500	0.720	Vigun06g120700	At4g23730	0.747
Vigun10g133400	At2g46070	0.717	Vigun04g175500	At1g73390	0.741
Vigun06g177400	At5g46240	0.717	**Vigun03g260900**	At4g38960	0.739
Vigun11g105000	At2g20740	0.716	Vigun09g134600	At4g16690	0.732
Vigun03g401700	At3g43220	0.711	Vigun06g053400	At4g26860	0.725
Vigun03g244200	At5g58140	0.711	Vigun01g069600	At3g27090	0.719
Vigun09g157700	At1g21400	0.708	Vigun07g189700	At3g17070	0.719
Vigun03g093300	At1g33811	0.707	Vigun11g152500	At5g59770	0.716
Vigun07g141800	At1g56280	0.706	Vigun04g185000	At3g01490	0.715
Vigun07g211600	At1g22750	0.706	Vigun03g279700	At5g67180	0.715
Vigun07g189700	At3g17070	0.705	Vigun10g133400	At2g46070	0.714
Vigun10g156700	At1g17280	0.704	Vigun03g244200	At5g58140	0.714
Vigun09g189700	At4g32850	0.703	Vigun03g401700	At3g43220	0.713
**Vigun05g174200**	At1g78510	0.701	Vigun06g177400	At5g46240	0.705
Vigun01g144200	At1g03950	0.700	**Vigun11g015600**	At5g06980	0.705
Vigun08g100400	At4g08920	0.700	Vigun09g189600	At4g2922	0.702
-	-	-	Vigun02g125700	At5g42990	0.702

r, Pearson correlation coefficient. IDs of cowpea *V. unguiculata* genes and their *A. thaliana* orthologs are from Phytozome v13 (Phytozome (doe.gov). **Emboldened gene IDs**, DEGs co-expressed with either *VunCCA1* and *VunLHY* or both

Supplemental Table 8 *VunLHY* variants in UCL mutant genome

Location	Sample Pattern	Affected Genes	Transcripts	Effects	
chrVu10: 37344133	0/0 1/1	Vigun10g153300	Vigun10g153300.1.v1.2	**TCG:** ref allele	**CTA:** upstream_gene_variant
			Vigun10g153300.2.v1.2	**TCG:** ref allele	**CTA:** upstream_gene_variant
			Vigun10g153300.4.v1.2	**TCG:** ref allele	**CTA:** upstream_gene_variant
			Vigun10g153300.6.v1.2	**TCG:** ref allele	**CTA:** upstream_gene_variant
			Vigun10g153300.7.v1.2	**TCG:** ref allele	**CTA:** upstream_gene_variant
			Vigun10g153300.8.v1.2	**TCG:** ref allele	**CTA:** upstream_gene_variant
			Vigun10g153300.9.v1.2	**TCG:** ref allele	**CTA:** upstream_gene_variant
				**TCG:** ref allele	**CTA:** intergenic_region
chrVu10: 37344604	0/0 1/1	Vigun10g153300	Vigun10g153300.1.v1.2	**CA:** ref allele	**TC:** upstream_gene_variant
			Vigun10g153300.2.v1.2	**CA:** ref allele	**TC:** upstream_gene_variant
			Vigun10g153300.4.v1.2	**CA:** ref allele	**TC:** upstream_gene_variant
			Vigun10g153300.6.v1.2	**CA:** ref allele	**TC:** upstream_gene_variant
			Vigun10g153300.7.v1.2	**CA:** ref allele	**TC:** upstream_gene_variant
			Vigun10g153300.8.v1.2	**CA:** ref allele	**TC:** upstream_gene_variant
			Vigun10g153300.9.v1.2	**CA:** ref allele	**TC:** upstream_gene_variant
				**CA:** ref allele	**TC:** intergenic_region

Supplemental Table 10 *VunBRI1* variants in UCL mutant genome

Location	Sample Pattern	Affected Genes	Transcripts	Effects	
chrVu02: 18476009	0/0 1/1	Vigun02g046500	Vigun02g046500.1.v1.2	**A:** ref allele	**T:** downstream_gene_variant
			Vigun02g046500.2.v1.2	**A:** ref allele	**T:** downstream_gene_variant
				**A:** ref allele	**T:** intergenic_region
chrVu02: 18476011	0/0 1/1	Vigun02g046500	Vigun02g046500.1.v1.2	**C:** ref allele	**T:** downstream_gene_variant
			Vigun02g046500.2.v1.2	**C:** ref allele	**T:** downstream_gene_variant
				**C:** ref allele	**T:** intergenic_region
chrVu02: 18483367	0/0 1/1	Vigun02g046500	Vigun02g046500.1.v1.2	**ACA:** ref allele	**GCC:** upstream_gene_variant
			Vigun02g046500.2.v1.2	**ACA:** ref allele	**GCC:** upstream_gene_variant
				**ACA:** ref allele	**GCC:** intergenic_region

Supplemental Table 9 *VunRVE4* variants in UCL mutant genome

Location	Sample Pattern	Affected Genes	Transcripts	Effects
chrVu07: 10891928	0/0 1/1	Vigun07g078900	Vigun07g078900.1.v1.2	**ATA:** ref allele **ATATATATATGTA:** downstream_gene_variant
			Vigun07g078900.2.v1.2	**ATA:** ref allele **ATATATATATGTA:** downstream_gene_variant
			Vigun07g078900.5.v1.2	**ATA:** ref allele **ATATATATATGTA:** downstream_gene_variant
			Vigun07g078900.3.v1.2	**ATA:** ref allele **ATATATATATGTA:** downstream_gene_variant
		Vigun07g079000	Vigun07g079000.1.v1.2	**ATA:** ref allele **ATATATATATGTA:** downstream_gene_variant
				**ATA:** ref allele **ATATATATATGTA:** intergenic_region

Supplemental Table 11 *VunLOB* variants in UCL mutant genome

Location	Sample Pattern	Affected Genes	Transcripts	Effects	
chrVu02: 29738970	0/0 1/1	Vigun02g150500	Vigun02g150500.1.v1.2	**G:** ref allele	**T:** downstream_gene_variant
				**G:** ref allele	**T:** intergenic_region
chrVu02: 29738992	0/0 1/1	Vigun02g150500	Vigun02g150500.1.v1.2	**A:** ref allele	**T:** downstream_gene_variant
				**A:** ref allele	**T:** intergenic_region

Note S1 GFFCompare accuracy statistics for UCL/wild-type/Vunguiculata_540_v1.2 StringTie-merged reference transcriptome annotation

```
# gffcompare v0.12.6 | Command line was:
#gffcompare -V -r reference_annotation -d 100 -p TCONS
Reference_transcriptome_annotation__StringTie_merge_output
#

#= Summary for dataset: Reference_transcriptome_annotation__StringTie_merge_output
#     Query mRNAs :   61283 in   33697 loci  (53289 multi-exon transcripts)
#            (11194 multi-transcript loci, ~1.8 transcripts per locus)
# Reference mRNAs :   54449 in   31862 loci  (48192 multi-exon)
# Super-loci w/ reference transcripts:    31654
#-----------------| Sensitivity | Precision  |
        Base level:   100.0     |    93.8    |
        Exon level:    99.1     |    94.5    |
      Intron level:   100.0     |    97.7    |
Intron chain level:   100.0     |    90.4    |
  Transcript level:    99.9     |    88.7    |
       Locus level:    99.8     |    93.9    |

     Matching intron chains:   48191
       Matching transcripts:   54373
              Matching loci:   31808

          Missed exons:       0/195070      (  0.0%)
           Novel exons:    3921/203154      (  1.9%)
        Missed introns:       1/150963      (  0.0%)
         Novel introns:    2007/154494      (  1.3%)
           Missed loci:       0/31862       (  0.0%)
            Novel loci:    2043/33697       (  6.1%)

 Total union super-loci across all input datasets: 33697
61283 out of 61283 consensus transcripts written in gffcmp.annotated.gtf (0 discarded as redundant)
```
